# Publisher Correction: TGF-β signaling in Th17 cells promotes IL-22 production and colitis-associated colon cancer

**DOI:** 10.1038/s41467-020-19685-x

**Published:** 2020-11-06

**Authors:** Laura Garcia Perez, Jan Kempski, Heather M. McGee, Penelope Pelzcar, Theodora Agalioti, Anastasios Giannou, Leonie Konczalla, Leonie Brockmann, Ramez Wahib, Hao Xu, Maria Carolina Amezcua Vesely, Shiwa Soukou, Babett Steglich, Tanja Bedke, Carolin Manthey, Oliver Seiz, Björn-Philipp Diercks, Stylianos Gnafakis, Andreas H. Guse, Daniel Perez, Jakob R. Izbicki, Nicola Gagliani, Richard A. Flavell, Samuel Huber

**Affiliations:** 1 I. Department of Medicine, University Medical Center Hamburg-Eppendorf, 20246 Hamburg, Germany; 2 Center for Immunobiology and Microbial Pathogenesis, Salk Institute for Biological Studies, 10010 La Jolla, CA USA; 3 Department of General, Visceral and Thoracic Surgery, University Medical Center Hamburg-Eppendorf, 20246 Hamburg, Germany; 4 Department of Microbiology and Immunology, Columbia University, New York, NY USA; 5 Department of Immunobiology, School of Medicine, Yale University, New Haven, CT 06520 USA; 6 Department of Biochemistry and Molecular Cell Biology, University Medical Center Hamburg-Eppendorf, 20246 Hamburg, Germany; 7 Laboratory of Innate Immunity, Department of Microbiology, Infectious Diseases and Immunology, Charité - Universitätsmedizin Berlin, Hindenburgdamm 30, 12203 Berlin, Germany; 8 Berlin Institute of Health (BIH), Anna-Louisa-Karsch Strasse 2, 10117 Berlin, Germany; 9 Mucosal and Developmental Immunology, Deutsches Rheuma-Forschungszentrum, Charitéplatz 1, 10117 Berlin, Germany; 10 Immunology and Allergy Unit, Department of Medicine, Solna, Karolinska Institute and University Hospital, Stockholm, Sweden; 11 Howard Hughes Medical Institute, School of Medicine, Yale University, New Haven, CT 06520 USA

**Keywords:** Lymphocyte differentiation, CD4-positive T cells, Tumour immunology

Correction to: *Nature Communications* 10.1038/s41467-020-16363-w, published online 25 May 2020

The original version of this article contained an error in Fig. 6d. During typesetting of the accepted paper, the dot plot for the third panel (0.5 µM) was duplicated into the second panel (0.1 µM). A correct version of Fig. 6 is shown below. The error has now been corrected in the original version of the article.

**Fig. 6:**
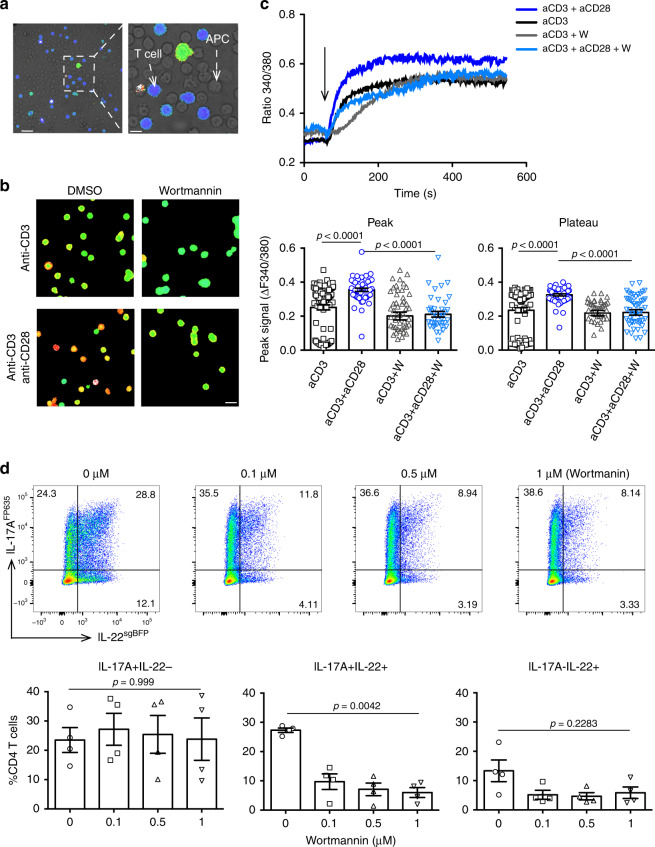
.

