# Supplementary material for: TGF-β signaling in Th17 cells promotes IL-22 production and colitis-associated colon cancer
Source: Nat Commun. 2020 May 25;11:2608. doi: 10.1038/s41467-020-16363-w (PMC7248087; doi:10.1038/s41467-020-16363-w)
Supplement: Supplementary file 1 — Supplementary Information [file 41467_2020_16363_MOESM1_ESM.pdf]

Perez et al.

TGF-β signaling in Th17 cells promotes IL-22 production and colitis associated colon cancer

Supplementary information

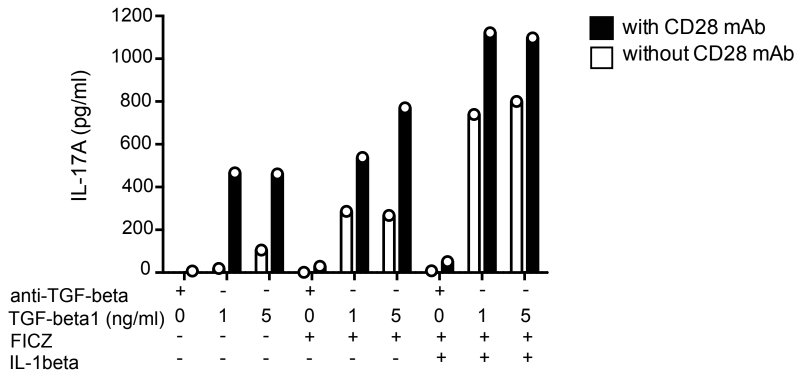

Supplementary Figure 1: TGF-β1 promotes IL-17A production *in vitro*.
Naïve T cells were isolated from spleen and lymph nodes of wild type (C57BL/6J) mice and cultured for four days in indicated conditions. IL-17A protein level was measured from culture supernatant (same as used in Figure 2a) by CBA, mean of technical duplicates is shown. Source data are provided as a Source data file.

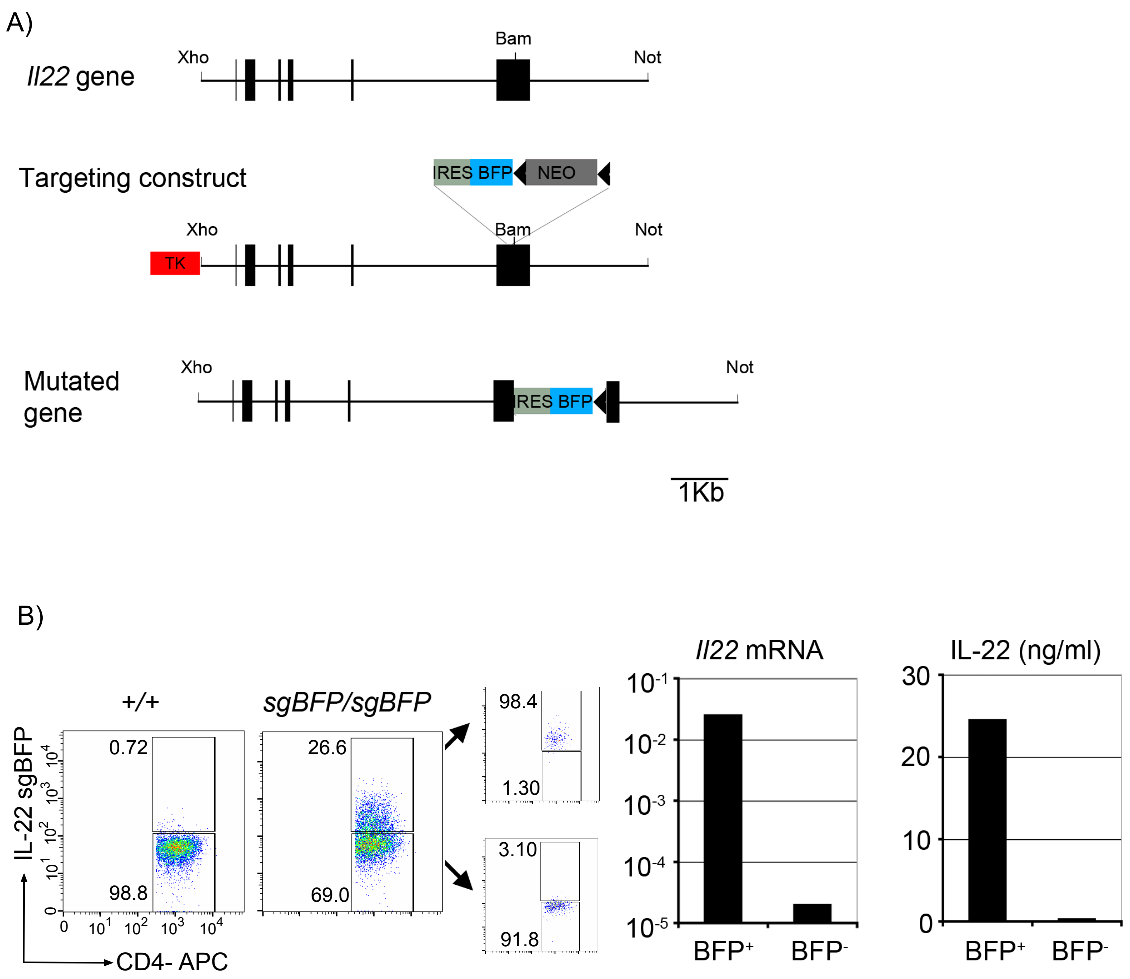

Supplementary Figure 2: Generation and validation of IL-22^{sgBFP} reporter mice.
A) Targeting construct. **B**) Naïve T cells were isolated from wild type and IL-22^{sgBFP} reporter mice and cultured in the presence of IL-6, IL-23, and TGF-β1 for four days. IL-22^{sgBFP} positive and negative cells were sorted using FACS. *Il22* mRNA expression was measured using RT-PCR. IL-22 protein levels were measured in cell culture supernatants of IL-22^{sgBFP} positive and negative CD4+ T cells upon restimulation for 48h using ELISA, mean of technical duplicates is shown. Results are representative of three independent experiments. Source data are provided as a Source data file.

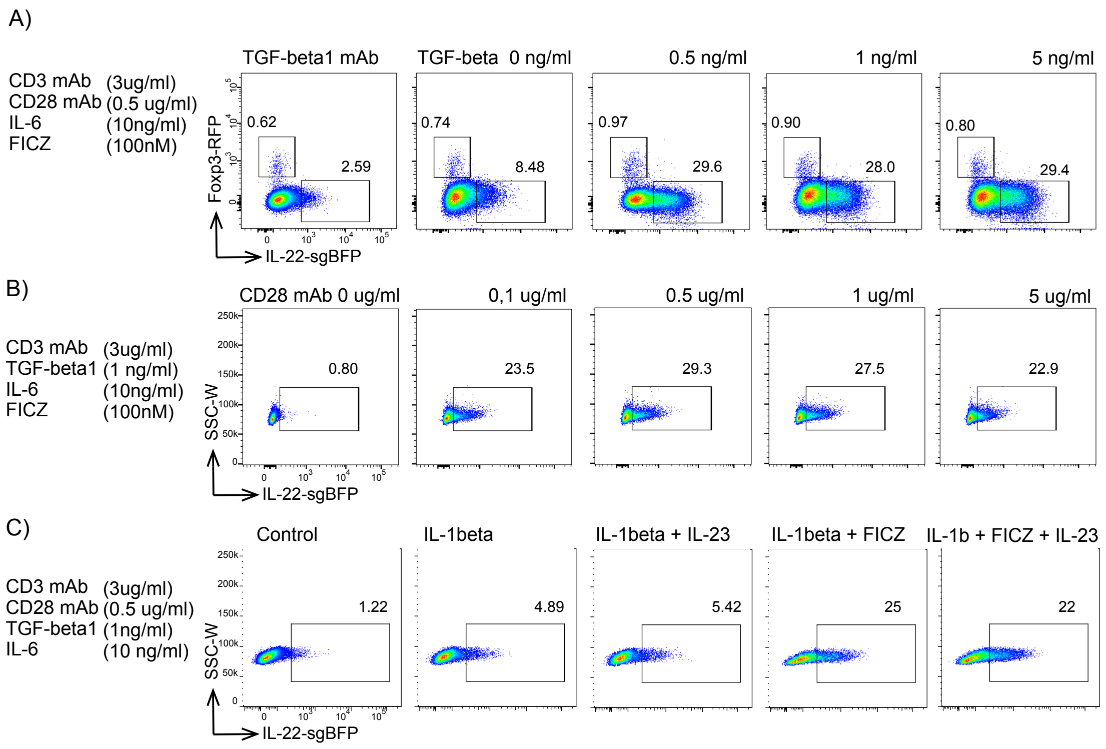

Supplementary Figure 3: Low dose of TGF-β1 and anti-CD28 is sufficient for IL-22 induction.

Naïve T cells were isolated from spleen and lymph nodes of Foxp3^{mRFP} x IL-17A^{eGFP} x IL-22^{sgBFP} reporter mice and cultured for four days in indicated conditions. **A**) Concentration of TGF-β1 was titrated for optimal IL-22 production *in vitro*. **B**) Concentration of CD28 mAb was titrated for optimal IL-22 production *in vitro*. **C**) Evaluation of the additive effect of IL-1β and IL-23 with FICZ in the production of IL-22 *in vitro*. Results are representative of two independent experiments.

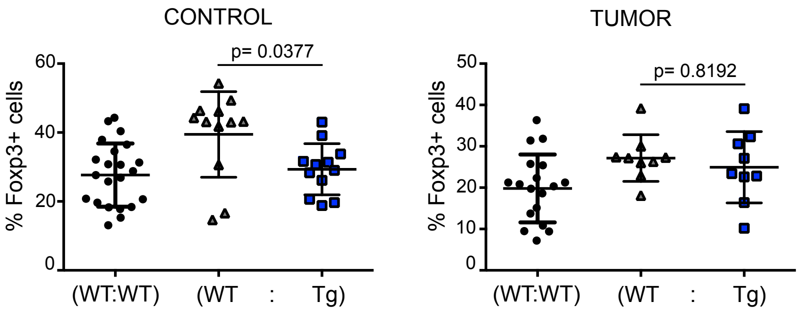

Supplementary Figure 4: The role of TGF-β signaling for the emergence of Foxp3+ T cells in colitis associated colorectal cancer.

CD4+ T cells from $Foxp3^{mRFP}$ x $IL\text{-}17A^{eGFP}$ x $IL\text{-}22^{sgBFP}$ (WT) or $Foxp3^{mRFP}$ x $IL\text{-}17A^{eGFP}$ x $IL\text{-}22^{sgBFP}$ x dnTGF-βR2 (Tg) mice were co-transferred into *Rag1-/-* prior to tumor induction. Frequency of Foxp3+ CD4+ T cells was analyzed by flow cytometry in both tumors and normal adjacent tissue (control). Results are cumulative from two independent experiments. Control: (WT:WT) n= 22; (WT:Tg) n= 12. Tumor: (WT:WT) n=18; (WT:Tg) =9. Lines indicate mean +/- sem. Two-sided Wilcoxon multiple comparisons test was performed (P<0.05) to assess the significance. Source data are provided as a Source data file.

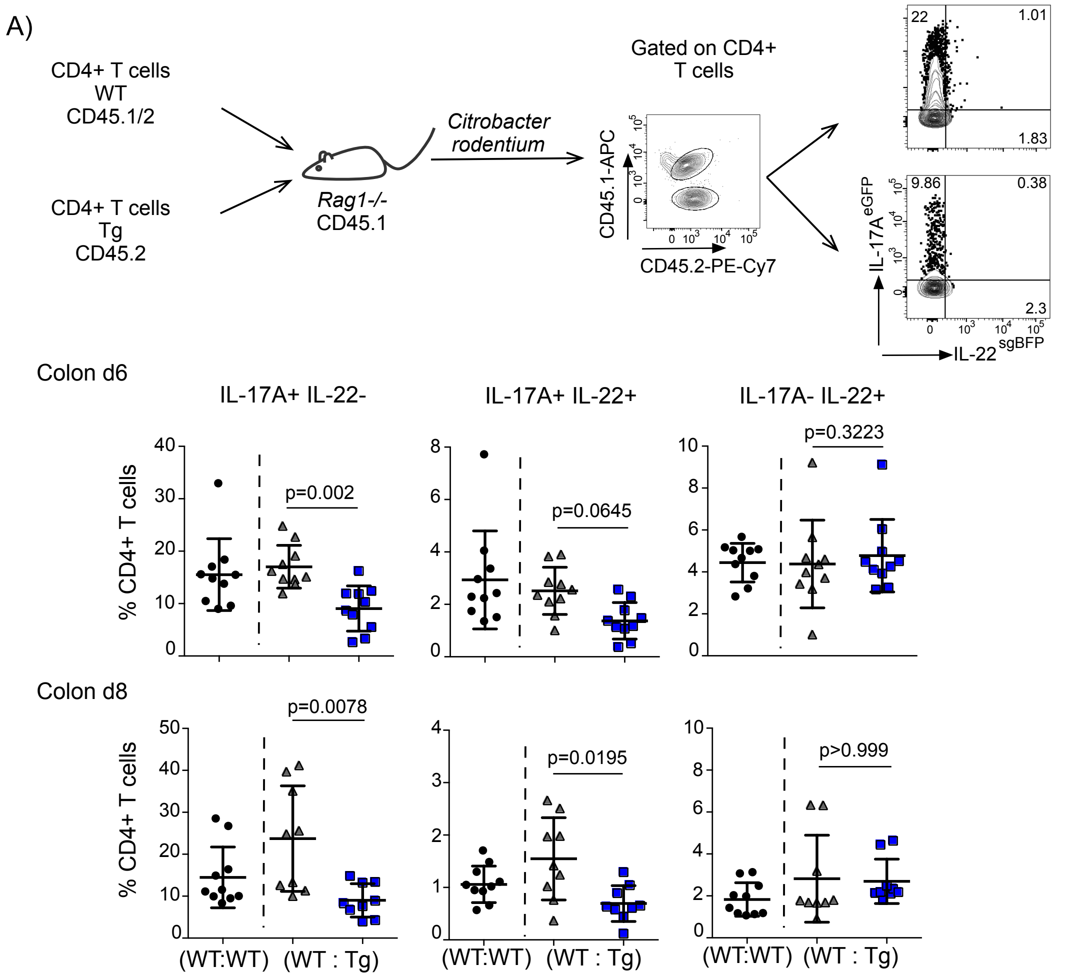

Supplementary Figure 5: TGF-β signaling in T cells promotes the emergence of IL-17+IL-22+ CD4+ T cells in *Citrobacter rodentium* infection in a direct manner.

A) CD4+ T cells from Foxp3^{mRFP} x IL-17A^{eGFP} x IL-22^{sgBFP} or Foxp3^{mRFP} x IL-17A^{eGFP} x IL-22^{sgBFP} x dnTGF-βR2 (Tg) mice were co-transferred into *Rag1-/-* prior infection. Production of IL-17 and IL-22 by T cells was analyzed by flow cytometry in the colon at day 6 and 8 post infection. Results are cumulative from two independent experiments. Colon day 6: (WT:WT) n= 10; (WT:Tg) n= 10. Colon day 8: (WT:WT) n= 10; (WT:Tg) n= 9 Lines indicate mean +/- sem. Two-sided Wilcoxon multiple comparisons test was performed (P<0.05) to assess the significance. Source data are provided as a Source data file.

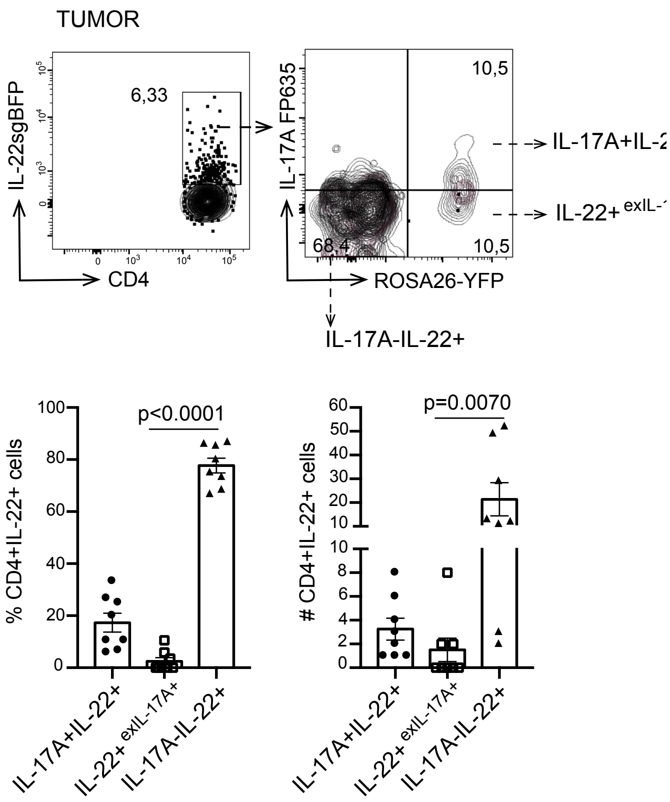

Supplementary Figure 6: Fate of IL-22 producing T cells in tumors.
Colitis associated colon cancer was induced in IL-17A^{Cre} x Rosa26^{YFP} x IL17A^{FP635} x IL-22^{sgBFP} mice. Lymphocyte isolation of tumors was performed and analyzed by flow cytometry. Frequency and cell number of indicated cell populations are shown. Each dot represents one mouse (n= 8). Bars represent mean, error bars show +/- sem. One-way ANOVA, Tukey's multiple comparisons test (P<0.05). Source data are provided as a Source data file.

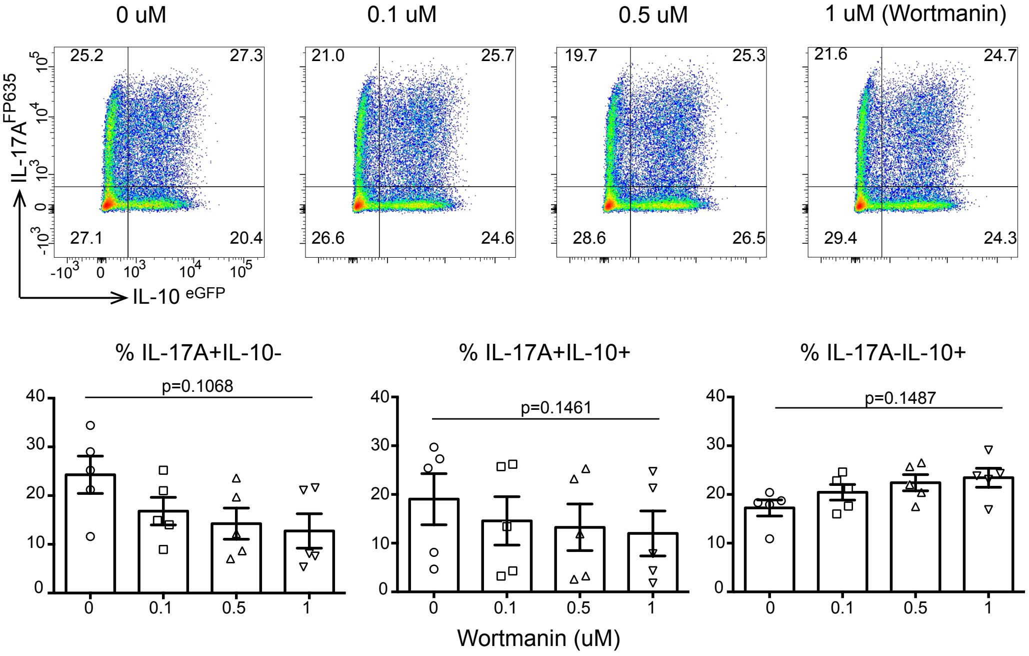

Supplementary Figure 7: PI3 Kinase activation is not essential for the emergence of IL-17+IL-10+ T cells.
Naïve CD4+ T cells from Foxp3^{mRFP} x IL-10^{eGFP} x IL-17^{FP635} x IL-22^{sgBFP} reporter mice were differentiated under Th17 polarizing conditions and increasing amounts of PI3K inhibitor (Wortmanin). Frequency of indicated cell populations are shown, n=4, bars represent mean, error bars show +/- sem. Data are cumulative from four independent experiments. One-way ANOVA, Dunnett's multiple comparisons test. Source data are provided as a Source data file.

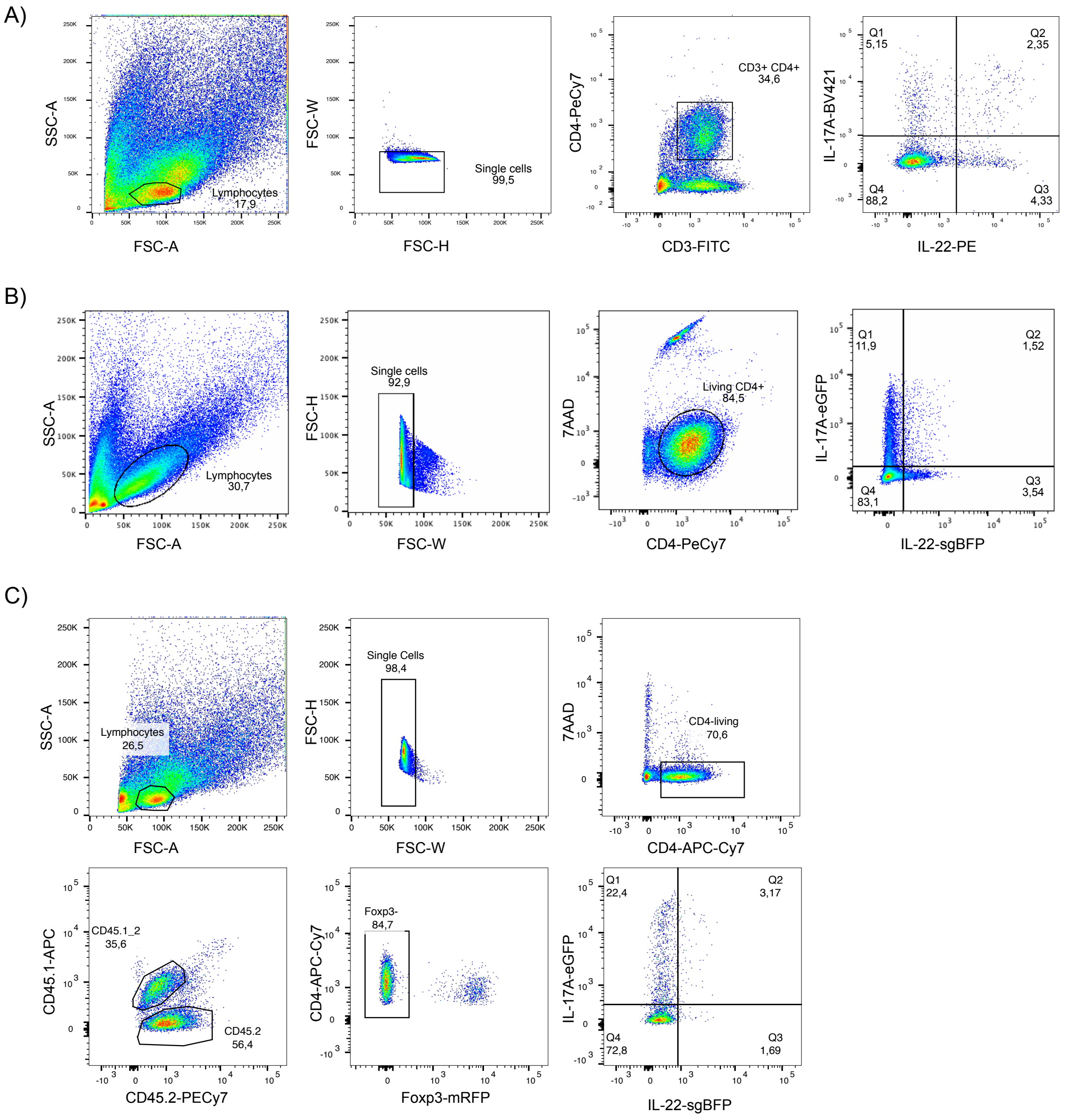

Supplementary Figure 8: Representative gating strategies in flow cytometry analysis.
A) Representative gating strategy of human flow cytometry analysis shown in Figure 1b. **B**) Representative gating strategy of flow cytometry analysis shown in Figure 2c, 5d and 6d. **C**) Representative gating strategy of flow cytometry analysis shown in Figure 3a and 4a.

Table S1. Patients characteristics

sex (M/F)	14/8
age (years)*	67 +/- 9.23

*mean +/- SD

Supplementary Table 1. Colorectal cancer patients characteristics
