# Supplementary material for: TGF-β signaling in Th17 cells promotes IL-22 production and colitis-associated colon cancer
Source: Nat Commun. 2020 May 25;11:2608. doi: 10.1038/s41467-020-16363-w (PMC7248087; doi:10.1038/s41467-020-16363-w)
Supplement: Supplementary file 3 — Reporting Summary [file 41467_2020_16363_MOESM3_ESM.pdf]

nature research

Corresponding author(s): Samuel Huber (s.huber@uke.de)

Last updated by author(s): Apr 6, 2020

Reporting Summary

Nature Research wishes to improve the reproducibility of the work that we publish. This form provides structure for consistency and transparency in reporting. For further information on Nature Research policies, see Authors & Referees and the Editorial Policy Checklist.

Statistics

For all statistical analyses, confirm that the following items are present in the figure legend, table legend, main text, or Methods section.

n/a	Confirmed	
☐	☒	The exact sample size (*n*) for each experimental group/condition, given as a discrete number and unit of measurement
☐	☒	A statement on whether measurements were taken from distinct samples or whether the same sample was measured repeatedly
☐	☒	The statistical test(s) used AND whether they are one- or two-sided<br>*Only common tests should be described solely by name; describe more complex techniques in the Methods section.*
☐	☒	A description of all covariates tested
☐	☒	A description of any assumptions or corrections, such as tests of normality and adjustment for multiple comparisons
☐	☒	A full description of the statistical parameters including central tendency (e.g. means) or other basic estimates (e.g. regression coefficient) AND variation (e.g. standard deviation) or associated estimates of uncertainty (e.g. confidence intervals)
☐	☒	For null hypothesis testing, the test statistic (e.g. *F*, *t*, *r*) with confidence intervals, effect sizes, degrees of freedom and *P* value noted<br>*Give P values as exact values whenever suitable.*
☒	☐	For Bayesian analysis, information on the choice of priors and Markov chain Monte Carlo settings
☐	☒	For hierarchical and complex designs, identification of the appropriate level for tests and full reporting of outcomes
☐	☒	Estimates of effect sizes (e.g. Cohen's *d*, Pearson's *r*), indicating how they were calculated

Our web collection on statistics for biologists contains articles on many of the points above.

Software and code

Policy information about availability of computer code

Data collection	Diva Software v.8, Volocity v.6.6.2
Data analysis	GraphPad Prism v.7, FlowJo v.10, Graphic v3.1, Openlab software v.5.5.2, FIJI Image J v.2.0.0

For manuscripts utilizing custom algorithms or software that are central to the research but not yet described in published literature, software must be made available to editors/reviewers. We strongly encourage code deposition in a community repository (e.g. GitHub). See the Nature Research guidelines for submitting code & software for further information.

Data

Policy information about availability of data

All manuscripts must include a data availability statement. This statement should provide the following information, where applicable:

- Accession codes, unique identifiers, or web links for publicly available datasets
- A list of figures that have associated raw data
- A description of any restrictions on data availability

We include a list of figures that have associated raw data

Field-specific reporting

Please select the one below that is the best fit for your research. If you are not sure, read the appropriate sections before making your selection.

☒ Life sciences ☐ Behavioural & social sciences ☐ Ecological, evolutionary & environmental sciences

For a reference copy of the document with all sections, see nature.com/documents/nr-reporting-summary-flat.pdf

Life sciences study design

All studies must disclose on these points even when the disclosure is negative.

Sample size	Sample size was chose based on previous work from our group with the same models. On the basis of these data we did a POWER analysis using Wilcoxon-Mann-Whitney test
Data exclusions	We excluded samples only for technical reasons, e.g. insufficient cell numberupon isolation procedure. No obtained data were excluded
Replication	All mouse experiments were done at least twice in independent experiments. Specifics are indicated in the figure legends. All attempts at replication were successful
Randomization	We used WT and transgenic mice, which were allocated to specific gropus based on their genotype. Thus there was no randomization
Blinding	The scoring of the experiments was done in a blinded fashion

Reporting for specific materials, systems and methods

We require information from authors about some types of materials, experimental systems and methods used in many studies. Here, indicate whether each material, system or method listed is relevant to your study. If you are not sure if a list item applies to your research, read the appropriate section before selecting a response.

Materials & experimental systems

n/a	Involved in the study	
☐	☒	Antibodies
☒	☐	Eukaryotic cell lines
☒	☐	Palaeontology
☐	☒	Animals and other organisms
☐	☒	Human research participants
☒	☐	Clinical data

Methods

n/a	Involved in the study	
☒	☐	ChIP-seq
☐	☒	Flow cytometry
☒	☐	MRI-based neuroimaging

Antibodies

Antibodies used	Mouse: CD4-PeCy7, RM4-5, Biolegend, B249467 (1:400); TCR-B-APC, H57-597, Biolegend, B160272 (1:400); CD45.1-APC, A20, Biolegend, B209251 (1:400); CD45.2-PECy7, 104, Biolegend, B214388 (1:400); CD4-APC-Cy7, GK1.5, Biolegend, B213762 (1:800); CD4-BV786, RM4-5, Biolegend, B236637 (1:1000); CD3-PE, 17A2, Biolegend, B210714 (1:800); CD4-BUV737, GK1.5, BD, 4324664 (1:800); CD45.2-AF700, 104, Biolegend, B252126 (1:200); CD8a APC-Cy7 ,53-6.7, Biolegend, B217172 (1:600); CD3e-BUV395, 17A2, BD, 8043925 (1:800).<br>Human: IL-17A-BV421, BL168, Biolegend, B192196 (1:200); IL-22-PE, 22URTI, eBioscience, E11018-1633 (1:200); TNF-a-BV605, MAb1, Biolegend, B191202 (1:200); INF-g-BV786, 4S.B3, Biolegend, B216665 (1:250); CD3-BUV737, UCHT1, BD, Biosciences, 7045559 (1:200); CD4-PECy7, OKT4, Biolegend, B189535 (1:600); CD45-PECy5, HI30, Biolegend, B215344 (1:400).
Validation	Mouse:<br>C57BL/6 mouse splenocytes were used to test CD4-PeCy7, TCR-B-APC, CD45.2-PECy7, CD4-APC-Cy7, CD4-BV786, CD3-PE, CD45.2-AF700, CD45.2-AF700, CD8a APC-Cy7 and CD3e-BUV395.<br>SJL mouse splenocytes were used to test CD45.1-APC<br>Mouse CTL clone V4 was used to test CD4-BUV737<br>Human:<br>Human peripheral blood lymphocytes, stimulated with PMA + Ionomycin for 6 hours (in the presence of monensin), stained with CD3 FITC, fixed and permeabilized were used to test IL-17A-BV421, TNF-a-BV605 and INF-g-BV786.<br>Human peripheral blood was used to test CD3-BUV737, CD4-PECy7 and CD45-PECy5.<br>Restimulated, Th17-polarized CD4+ normal human peripheral blood cells or stimulated total normal human peripheral blood cells were used to test IL-22-PE.

Animals and other organisms

Policy information about studies involving animals; ARRIVE guidelines recommended for reporting animal research

Laboratory animals	Mus musculus: Rag1-/- knock out mice, Il22-/- knock out mice, dnTGF-βR2 transgenic mice, TGFBR2fl/fl mice, IL-17ACre mice, Foxp3mRFP reporter mice, IL-17AeGFP reporter mice, IL-17AFP635 reporter mice, IL-10eGFP reporter mice and IL-22sgBFP reporter mice.<br>Males and females age and sex macthed littermates between 4-16 weeks were used.
Wild animals	The study did not involved wild animals

Field-collected samples	The study did not involve field-collected samples
Ethics oversight	All animals were cared for in accordance with the institutional review board 'Behörde für Soziales, Familie, Gesundheit und Verbraucherschutz' (Hamburg, Germany) and Yale University

Note that full information on the approval of the study protocol must also be provided in the manuscript.

Human research participants

Policy information about studies involving human research participants

Population characteristics	We analyzed normal and adjecent tissue from the same patient. Population characteristics: 14 males and 8 females, average age is 67 +/- 9,23 years old.
Recruitment	Patients were recreuited from the surgical deparment prior to operation due to colorectal cancer. We only recruited patients that did not undervent radio-chemotherapy to avoid a potential bias due to the therapy
Ethics oversight	All human studies were approved by the ethical committee (Ethik-Kommission der Aerztekammer Hamburg)

Note that full information on the approval of the study protocol must also be provided in the manuscript.

Flow Cytometry

Plots

Confirm that:

☒ The axis labels state the marker and fluorochrome used (e.g. CD4-FITC).

☒ The axis scales are clearly visible. Include numbers along axes only for bottom left plot of group (a 'group' is an analysis of identical markers).

☒ All plots are contour plots with outliers or pseudocolor plots.

☒ A numerical value for number of cells or percentage (with statistics) is provided.

Methodology

Sample preparation	Cells were isolated from human and mouse intestinal tissues and stained in MACS Buffer (1xPBS, 1%FBS, 0,5% EDTA)
Instrument	LSR Fortessa II
Software	Diva Software
Cell population abundance	The purity of the FACS-sorted populations was around 95%. To confirm the purity, the samples were analyzed again by flow cytometry after sorting
Gating strategy	SSC-A vs FSC-A; FSC-H vs FSC-W; Live/dead staining vs CD4; IL-17A vs IL-22

☒ Tick this box to confirm that a figure exemplifying the gating strategy is provided in the Supplementary Information.
